# A first look at the metabolic rate of Greenland sharks (*Somniosus microcephalus*) in the Canadian Arctic

**DOI:** 10.1038/s41598-020-76371-0

**Published:** 2020-11-09

**Authors:** Eric Ste-Marie, Yuuki Y. Watanabe, Jayson M. Semmens, Marianne Marcoux, Nigel E. Hussey

**Affiliations:** 1 Department of Integrative Biology, University of Windsor, Windsor, ON N9B 3P4 Canada; 2 National Institute of Polar Research, Tachikawa, Tokyo 190-8518 Japan; 3 Department of Polar Science, The Graduate University for Advanced Studies, SOKENDAI, Tachikawa, Tokyo 190-8518 Japan; 4 Fisheries and Aquaculture Centre, Institute for Marine and Antarctic Studies, University of Tasmania, Taroona, TAS 7053 Australia; 5 Arctic and Aquatic Research Division, Fisheries and Oceans Canada, 501 University Crescent, Winnipeg, MB R3T 2N6 Canada

**Keywords:** Ecology, Ecophysiology

## Abstract

Metabolic rate is intricately linked to the ecology of organisms and can provide a framework to study the behaviour, life history, population dynamics, and trophic impact of a species. Acquiring measures of metabolic rate, however, has proven difficult for large water-breathing animals such as sharks, greatly limiting our understanding of the energetic lives of these highly threatened and ecologically important fish. Here, we provide the first estimates of resting and active routine metabolic rate for the longest lived vertebrate, the Greenland shark (*Somniosus microcephalus*). Estimates were acquired through field respirometry conducted on relatively large-bodied sharks (33–126 kg), including the largest individual shark studied via respirometry. We show that despite recording very low whole-animal resting metabolic rates for this species, estimates are within the confidence intervals predicted by derived interspecies allometric and temperature scaling relationships, suggesting this species may not be unique among sharks in this respect. Additionally, our results do not support the theory of metabolic cold adaptation which assumes that polar species maintain elevated metabolic rates to cope with the challenges of life at extreme cold temperatures.

## Introduction

Organisms inhabiting extreme environments have long been of special interest to ecologists, physiologists and evolutionary biologists alike^1,2^, particularly as these environments, including the poles, deserts and the deep sea are not rare, but in fact cover vast expanses of the planet^3^. To assess the mechanisms facilitating life in extreme environments, the study of metabolic rate is regarded as a powerful tool given it combines insight into both the physiology and ecology of an organism^4,5^. This is based on the premise that the rates at which lifeforms acquire and expend energy are intricately linked to the abiotic and biotic conditions that constrain individual life on a daily basis^6^. In ectotherms, body mass (biotic) and environmental temperature (abiotic) are amongst the most studied variables known to influence metabolic rate^6–8^. Since Kleiber first published his seminal work linking body mass to metabolic rate^9^, much research has focused on defining this relationship within and across taxonomic boundaries^7,10,11^. Although the exact extent to which metabolic rate changes with the mass of organisms can vary^12^, the general pattern that mass-adjusted metabolic rate decreases with increasing body mass is widely observed and accepted as a fundamental biological concept^6^.

Temperature’s effect on metabolic rate, similar to that of mass, can be assessed across species (i.e. interspecific^7^), as well as within species (i.e. intraspecific^13^). While intraspecific scaling relationships can be useful when modeling the energetic needs of a specific animal under natural conditions^4^, interspecific relationships are useful as a reference point for the comparison of species^14^. Understanding these patterns is important since changes in energetic demand have been shown to systematically impact behaviour, life history (e.g. longevity, age at maturity, reproductive periodicity), and feeding requirements of individuals, which in turn affect population dynamics and ecosystem function^6^. Furthermore, unique data for extreme-temperature adapted species can broaden the scope and confidence of interspecific metabolic scaling relationships that aid in the development of ecologically relevant bioenergetic and evolutionary hypotheses^10^. For example, it has long been argued that species adapted to polar environments maintain relatively elevated metabolic rates to enable physiological processes that would otherwise be hindered by the extreme cold temperatures they inhabit^15^. In other words, a polar species is expected to have a metabolic rate that is higher than that predicted by the interspecies scaling relationship for a given temperature^16^. While data from more recent studies contradict this theory, demonstrating that polar species are not metabolically cold adapted^7,17–19^, a few studies also provide support for the theory^14^, indicating further investigation is needed across a wider phylogenetic range.

In fish, standard metabolic rate (SMR) is a fundamental measure of metabolism. It describes the basic energetic maintenance costs of an unfed individual at rest. For many species, SMR cannot be feasibly estimated under laboratory or field conditions, so resting routine metabolic rate (rRMR) is often used as a proxy^20^. This metric generally describes the same conditions as SMR, but is used when the latter’s strict assumptions cannot be met (e.g. if the fish exhibits minor postural fin movements during respirometry trials). Measuring SMR (or rRMR) poses additional challenges when studying sharks, as respirometry trials are expensive and logistically difficult to perform on large bodied individuals^21^. As such, SMR estimates for sharks are relatively rare and often skewed towards small species and juveniles^13,22^. In addition, active metabolic rates are often used to extrapolate SMR in obligate ram-ventilating species^4^, which can lead to variable estimates depending on the methodology used and the range of swim speeds covered^20^. Recent studies have found creative ways to curtail some of these challenges^23,24^, but overall, the metabolic rates of sharks remain relatively understudied.

The Greenland shark (*Somniosus microcephalus*) is one of the largest carnivorous fish species that is widely distributed across the North Atlantic and Arctic oceans, yet many aspects of its physiology and ecology remain a mystery including its metabolic rate^25,26^. While previous work has used dynamic energy budget (DEB) models to estimate certain life history characteristics in this species (e.g. gestation period), these have yet to be validated experimentally^27^. Greenland sharks occur at higher latitudes than all known species of shark and, as such, experience some of the coldest water temperatures on the planet (as low as −1.8 °C^25^). At adult lengths reaching greater than 5 m and with an estimated lifespan of 392 ± 120 years, they are the largest fish inhabiting the Arctic and the oldest known vertebrate species on the planet^25,28^. Paradoxically, they are also among the slowest fish in the ocean when accounting for body size, with a maximum recorded swim speed of only 0.74 m·s^−1^^29^. Despite the obvious uniqueness of Greenland sharks, their size and tendency to inhabit deep and remote areas of the ocean has made studying them expensive and logistically difficult^26^. Even so, their relatively high trophic position (4.2–7.7^30^) and abundance (up to 15.5 individuals per km^2^ ^31^) imply that they are important top-down regulators in Arctic food webs. In addition, through the scavenging of large carcasses (e.g. whale falls), Greenland sharks contribute to nutrient cycling which could aid in stabilizing food webs^32^.

Drawing from a novel dataset comprised of oxygen consumption rates measured through field respirometry trials, we provide the first estimates of resting and active routine metabolic rate (rRMR and aRMR) for the Greenland shark. Representing an extreme in terms of both body size and experimental temperature, we integrate our estimates with those of all sharks studied to date to derive a shark-specific interspecies metabolic scaling relationship for mass and temperature. We then compare our metabolic rate estimates for Greenland sharks with the values predicted by this derived equation in order to test for metabolic cold adaptation in this species. As a large and slow-moving species inhabiting extreme low temperatures, and given that most recent work has found little evidence supporting metabolic cold adaptation in polar species^19^, we hypothesized that Greenland sharks have predictably low metabolic rates when compared to all other sharks studied to date^7,13,33^.

## Results

### Respirometry

Using two large custom-built field respirometers, we measured the metabolic oxygen consumption rates of four Greenland sharks with individuals reaching body masses exceeding those used in previous studies on other fish (33–126 kg; Table 1). The largest of these individuals, held in a 16,570 L swimming pool in the high Arctic (Tremblay Sound, Nunavut), had an estimated mass that was more than double that of the largest shark previously studied in a respirometer (Previous record = 47.7 kg^23^). Both resting and active routine metabolic rate (rRMR and aRMR) were estimated for this individual at an experimental temperature of 3.8 °C. Average mass-adjusted rRMR across measurement intervals for this shark was 23.07 ± 4.62 (SD) mgO_2_h^−1^ kg^−0.84^, while aRMR during an approximate twenty-minute period when the shark swam volitionally with a constant tailbeat frequency (TBF) of 0.18 Hz was 30.96 mgO_2_h^−1^ kg^−0.84^. Of the three individuals studied using a smaller rectangular respirometer aboard the MV Kiviuq II the following year (Scott Inlet, Nunavut), two were inactive for extended periods providing estimates of rRMR of 22.29 ± 2.90 and 17.23 ± 0.90 (SD) mgO_2_h^−1^ kg^−0.84^, at 4.9–5.1 °C. The third individual remained active throughout the trial yielding an aRMR estimate of 40.46 ± 2.17 (SD) mgO_2_h^−1^ kg^−0.84^, at 4.9 °C; however, this individual’s movement was inhibited by the holding tank, so we excluded it from further analysis.

**Table 1:** Summarized data for Greenland sharks (*Somniosus microcephalus*) that underwent respirometry trials in Tremblay Sound and Scott Inlet, Nunavut, Canada (n = 4). Reported study temperatures represent the mean recorded temperature throughout the trials.

Mean mass-adjusted metabolic rate (mgO_2_ h^−1^ kg^−0.84^)										
Shark ID	Sex	FL (cm)	Mass (kg)	Date	Location	Study temp (℃)	rRMR ± SD	aRMR ± SD	TBF (Hz)	Respirometer
1	M	227	126^a^	2018-09-01	Tremblay Sound	3.8	23.07 ± 4.62	30.96 ± NA	0.18	Circular
2	F	163	40.8^a^	2019-09-20	Scott Inlet	4.9	22.29 ± 2.90	–	–	Rectangular
3	F	172	52.4	2019-09-21	Scott Inlet	4.9	–	40.46 ± 2.17	0.23^b^	Rectangular
4	F	155	33.4	2019-09-21	Scott Inlet	5.1	17.23 ± 0.90	–	–	Rectangular
Mean	–		63.1	–	–	4.7	20.86 ± 3.17	–	–	–

^a^Mass estimated from Leclerc’s 2012 equation using fork-length (FL).

^b^Movement was restricted by wall of respirometer.

### Metabolic scaling in sharks

**Figure 1:**
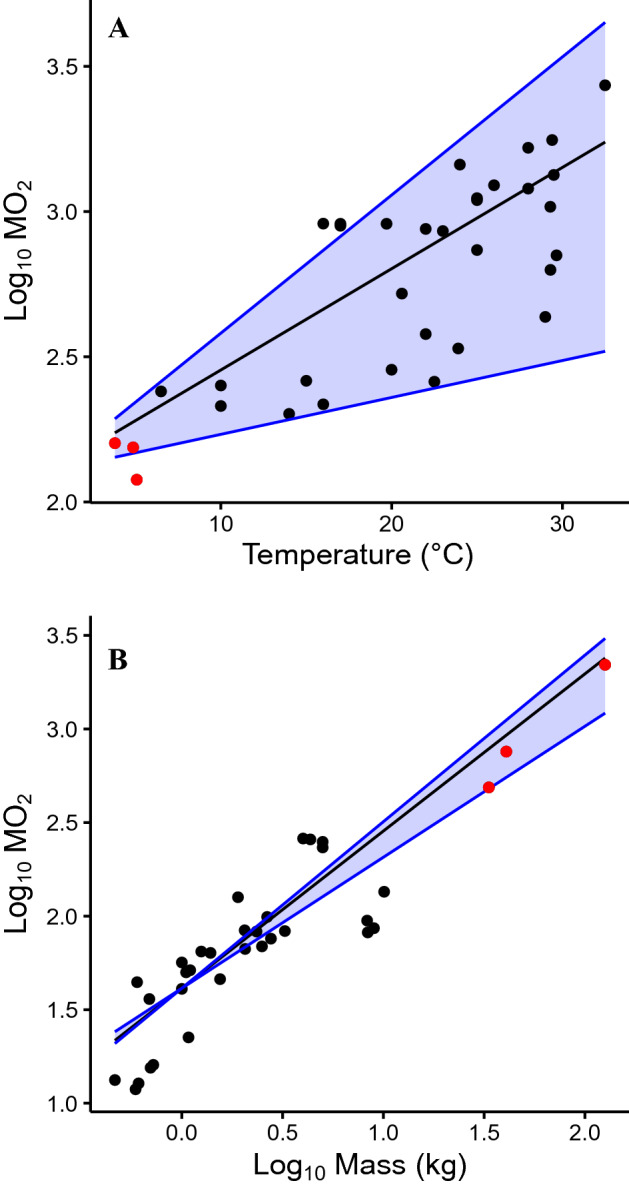
Comparison of literature derived whole-animal SMR and rRMR estimates for eighteen ectothermic shark species from nine families (MO_2_ units = log_10_ [mgO_2_ h^−1^]). Each black point represents the study-specific mean whole-animal MO_2_ provided for a species at a specific experimental temperature and mass. Red points represent rRMR estimates for the Greenland shark (*Somniosus microcephalus*). (**A**) Depicts log_10_-transformed SMR and rRMR estimates (adjusted to a standard mass of 10 kg) against experimental temperature. The black line represents the SMR of a shark species predicted using the interspecies Q_10_ value derived from our multiple regression analysis, while the blue lines represent the maximum and minimum Q_10_ values observed for specific shark species (*Ginglymostoma cirratum* and *Sphyrna lewini* respectively). (**B**) Depicts log_10_-transformed SMR and rRMR estimates (adjusted to a standard temperature of 10 °C) against the log_10_-transformed mean mass of sharks used in each study. The black line represents the SMR of a shark species predicted using the interspecies allometric scaling exponent derived from our multiple regression analysis, while the blue lines encompass the range of predicted SMR values calculated with commonly used allometric scaling exponents derived for global teleost fish in previous meta-analyses (see “Methods”).

From previous experimental studies, we extracted rRMR and SMR estimates for eighteen shark species spanning nine families (SI, Table S1). These experimental studies were conducted on animals ranging in size from < 0.5 to 12.4 kg and in experimental temperatures from 6.5 to 32.5 °C. Combining estimates with our rRMR results for Greenland sharks, we derived interspecific mass and temperature scaling coefficients for whole-animal metabolic rate via multiple regression analysis with each species weighted evenly (adjusted R^2^ = 0.761, n = 34, p < 0.0001; Fig. 1). The resulting mass coefficient translates to an allometric scaling exponent of 0.84, whose 95% confidence intervals (0.67–1.01) include the range of values published for global teleost fish (0.70–0.89^7,11,34^). The coefficient describing the effect of temperature on log_10_ metabolic rate (0.035) can be approximated by an overall interspecific Q_10_ of 2.23 across a ~ 29 °C temperature range (3.8–32.5 °C). This interspecific Q_10_ is within the wide range of intraspecific values derived for individual shark species (1.34–2.99; full Q_10_ list provided in SI Table S2), and its 95% confidence intervals include both the overall interspecific Q_10_ and median intraspecific Q_10_ values derived for teleost fish (1.83 and 2.40^7^). Additionally, we found that the rRMR estimates for the Greenland sharks studied here were all within the confidence intervals predicted by our overall interspecies metabolic scaling model.

## Discussion

Our whole-animal rRMR results for Greenland sharks indicate that these fish have very low energetic needs. However, the rRMR of examined Greenland sharks is well within the 95% confidence intervals predicted by our interspecific metabolic scaling relationship for mass and temperature across sharks. As such, our findings suggest that Greenland sharks are not metabolically cold adapted. While these results present a preliminary look at the metabolic ecology of this species, further investigation into the effect of mass and temperature on metabolic rate across individual Greenland sharks is required to accurately predict the dynamics of metabolic rate for this species in the wild.

The analysis of resting metabolic rate across shark species provided an allometric scaling exponent that was comparable to those derived for teleost species (0.70–0.89^7,11,34^). Due to the limited number of studies reporting respirometer derived SMR or rRMR estimates for sharks, and variability in the methods used to acquire these estimates^35^, we could not be as stringent with our study selection criteria as those used in previous analyses of teleosts. Nevertheless, our results identify that allometric scaling of metabolic rate in sharks across a large mass spectrum falls within the range of values for teleost fish examined at a global scale. However, scaling exponents for individual shark species, similar to teleost fish, will likely differ from the interspecific value according to lifestyle (e.g. pelagic vs. benthic), metabolic level, and swimming style of the species in question^34^. These factors vary immensely across shark species and can likely explain some of the variation observed around our interspecies scaling relationship. For example, the nurse shark (*Ginglymostoma cirratum*) has the lowest mass and temperature adjusted SMR among studied shark species because it is adapted to a relatively inactive lifestyle^36^. So far, only two studies have assessed intraspecific metabolic allometry in sharks, both of which yielded similar scaling exponents to our overall interspecific value of 0.84 (0.86 for lesser spotted dogfish [*Scyliorhinus canicula*]^37^; 0.80 for zebra sharks [*Stegostoma fasciatum*]^23^).

Allometric scaling of metabolic rate is often used to estimate the energy requirements of large sharks (e.g. white shark [*Carcharodon carcharis*]^38^), yet most respirometry is conducted on small species^39^, or juveniles of large species which could have metabolic rates that differ from their adult counterparts^40,41^. When extrapolating the metabolic rates of large individuals using estimates derived for individuals that are order(s) of magnitude smaller, minor differences among commonly used scaling exponents can lead to large discrepancies in estimated results^21,23^. For example, extrapolated metabolic rates for whale sharks weighing 5000 kg varied by a factor of 6.5 depending on the scaling exponent used^23^. This example, albeit extreme, emphasizes the need for metabolic rate data for large-bodied sharks, thus reducing the need for extrapolation. In the absence of such information, studies attempting to model the energetics of wild sharks typically rely on interspecific scaling equations or those borrowed from other species^38,42,43^, which undoubtedly increases the uncertainty surrounding estimates.

As with mass, the effect of temperature on metabolic rate is known to vary across species (SI Table S2). Several studies have addressed temperature dependent intraspecific scaling of metabolic rate in sharks, with metabolic Q_10_ estimates ranging from 1.34 in scalloped hammerhead sharks (*Sphyrna lewini*^22^) to 2.99 in nurse sharks (*Ginglymostoma cirratum*^33^). Due to our limited sample size and narrow range of experimental temperatures across respirometry trials, we could not address intraspecific scaling in Greenland sharks. However, the addition of metabolic oxygen consumption data for this cold-living species to the pooled data for all studied sharks, allowed for the assessment of how metabolic rate scales with temperature interspecifically across this diverse group of cartilaginous fish. We report that the across-shark Q_10_ of 2.23 is slightly higher than that derived for teleost fish (Q_10_ = 1.83) across a similar range of temperatures^7^. This could mean that, overall, the metabolic rates of sharks are more sensitive to temperature than those across teleost fish; however, the broad confidence intervals surrounding our Q_10_ estimate (1.74–2.85) include the value published for teleost fish, suggesting this small difference may not represent a real evolutionary difference between both groups of fish.

Conducting field respirometry trials on Greenland sharks in remote regions of the Arctic presents many logistical and methodological challenges. While the results of the present study provide novel insight into the metabolism of a large Arctic shark, several caveats must be acknowledged. Notably, short acclimation periods (2.5 h) prior to conducting respirometry trials could have led to inflated rRMR estimates arising from stress/recovery costs^20^. Additionally, we were unable to confirm if individuals were in a post-absorptive state, consequently specific dynamic action (SDA) could have increased the rate of oxygen uptake in our experimental animals if they were actively digesting a meal at the time of study^20,44^. Though important to consider, fasting a large polar ectotherm such as the Greenland shark could take weeks and would not have been feasible under field conditions. Even if the rRMR estimates provided here represent an over-estimate of the true SMR of Greenland sharks, we found no indication that Greenland sharks are metabolically cold-adapted. Given the methodological caveats outlined above, their true SMR might actually be lower than expected for a species inhabiting the extreme cold waters of the Arctic. The latter point would seem feasible given their longevity and proclivity for deep-sea environments, both of which have been linked to reduced metabolic rates in other fish^45,46^.

Despite having a seemingly unremarkable mass and temperature adjusted metabolic rate in comparison to other sharks, it is important to consider the implications of the extremely low whole-animal metabolic rates measured here at ecologically relevant experimental temperatures, as it relates to the ecological role of Greenland sharks in the Arctic. With such low energetic needs, Greenland sharks may be capable of surviving extended periods of time without feeding following the consumption of energy rich prey^26^. For example, assuming an assimilation efficiency of 73%^47^, and that 1 mol O_2_ is equal to 434 kJ^48^, the aRMR of the 126 kg shark studied in the Tremblay Sound respirometer would translate to a daily caloric requirement of only 192 kcal. If we further assume Greenland sharks can store energy in their tissues or as undigested food in their gut^49^, the consumption of a whole juvenile seal weighing 25 kg could theoretically allow the shark to survive > 365 days without subsequent feeding events (caloric value of ringed seal taken from^50^). This preliminary estimate accepts that aRMR measured at a specific activity level and temperature is not necessarily representative of the individual’s field metabolic rate but serves to contextualize its low metabolic rate in ecological terms.

Furthermore, the consumption of large meals by Greenland sharks combined with their slow metabolism could facilitate life in resource scarce environments, such as the Arctic, and allow this species to maximize the use of seasonally abundant or migratory prey such as marine mammals. This is further supported by evidence that a fish’s tendency to exhibit hyperphagia increases dramatically at cold temperatures^51^. However, the mechanisms driving the feeding and digestive physiology of wild free roaming animals remain largely unknown and understudied, making it difficult for ecologists to explain or predict feeding behaviour in the wild. As such, energetic models for animals under natural conditions require significant assumptions^49^. Further study of the digestive physiology and field metabolism of Greenland sharks is necessary to increase our understanding of hyperphagia and feeding frequency in this highly vulnerable species.

Among the world’s largest fish and inhabiting some of the deepest and coldest waters on the planet, the long-lived Greenland shark provides a unique model to study animal physiology under extreme conditions. Despite this, our results suggest the Greenland shark’s resting metabolic rate is unremarkable when the effects of temperature and mass are accounted for, but further investigation is needed to uncover how metabolic rate scales within the species. The logistics of measuring the metabolic rates of large sharks continues to prevent the widespread application of standardized respirometry practices commonly used to assess the metabolism of small fish. Despite this, we show that interspecific metabolic scaling with mass and temperature across sharks yields similar scaling coefficients as those derived for teleost fish, even with the inclusion of data for sharks at much larger body sizes than previously studied. As the use of metabolic data in ecological modeling grows in popularity, there is a pressing need to improve our understanding of the dynamics of metabolic rate within and across shark species.

## Methods

### Respirometry

**Figure 2:**
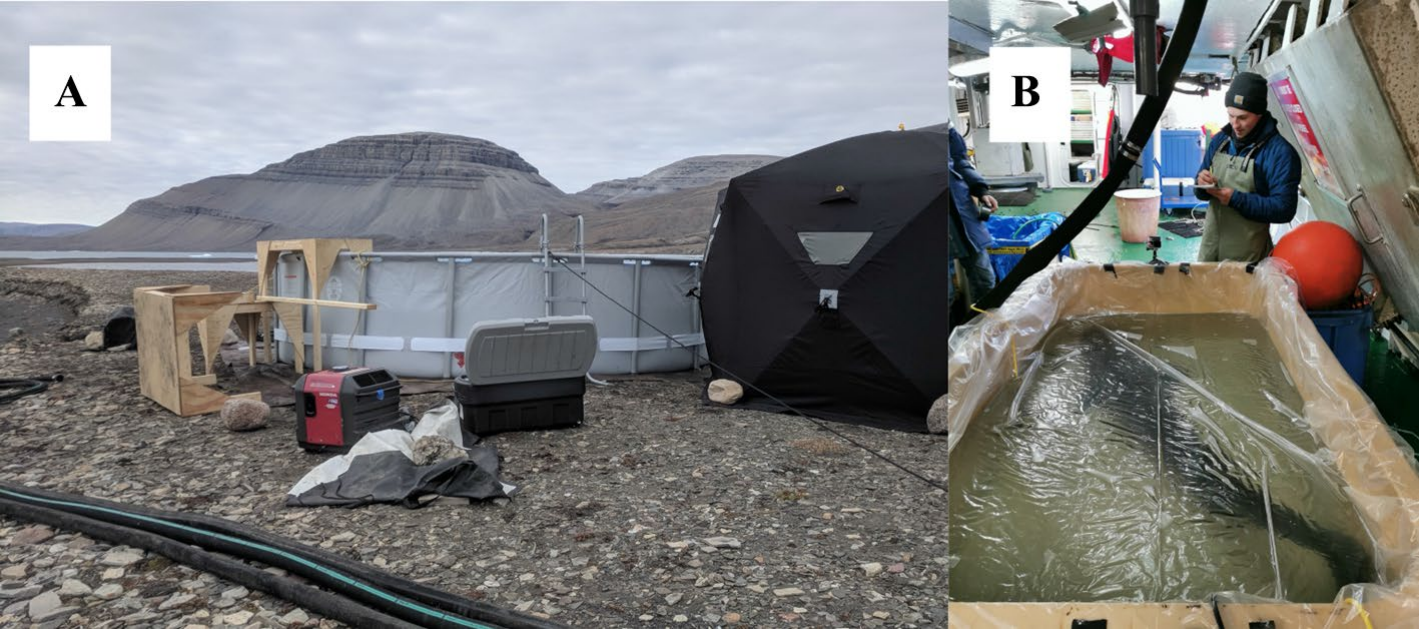
Photographs of the two respirometers used in this study. (**A**) Depicts the large “circular” type respirometer used in Tremblay Sound in 2018 (Photo by Eric Ste-Marie). (**B**) Depicts the smaller “rectangular” type respirometer used in Scott Inlet in 2019 (Photo of E. Ste-Marie taken by Jena Edwards and used with permission).

To estimate the metabolic demands of Greenland sharks, respirometry trials were conducted on temporarily captive wild sharks (see SI Appendix for details on all fishing and fieldwork protocols). We built two types of respirometer for this study (Fig. 2). The first was a 16,570 L circular static respirometer in Tremblay Sound that allowed the measurement of the routine oxygen consumption rate of a shark at rest and while swimming volitionally (i.e. rRMR and aRMR). The second was a smaller (600–910 L) rectangular tank in which we were able to measure the rRMR of sharks aboard a commercial fishing vessel (MV Kiviuq II) in Scott Inlet. Submersible pumps were used to homogenize dissolved oxygen levels in both respirometers during trials and plastic drop sheeting was used to seal the water surface area to prevent gas exchange with air. Due to the logistical challenges of conducting respirometry trials on large animals in the field and the assumed slow digestion rate of Greenland sharks at low temperatures, we could not starve individuals ahead of measuring their oxygen consumption rates. As such, we refer to our estimates as routine metabolic rate instead of true standard or active metabolic rate according to Chabot et al.^20^.

In both experimental setups, trials for each shark began after an acclimation period of 2.5 h at the same water temperatures recorded during the trials themselves (i.e. 3.7–3.8 °C in Tremblay Sound and 4.9–5.1 °C in Scott Inlet). Three to five 60-min trials were run intermittently for each shark in Scott Inlet (individual trial estimates available in SI Table S3), with twenty-minute intervals between each trial to replenish dissolved oxygen levels in the tank. Due to the large volume of the Tremblay Sound respirometer, dissolved oxygen levels remained high (> 95% original concentration) so only one depletion was performed. Timed notes were taken to track behavioural changes of individuals (i.e. swimming, resting, rolling) throughout each trial in both setups, allowing the selection of periods of continuous rest to estimate rRMR and periods of sustained swimming to estimate active routine metabolic rate (i.e. aRMR). Background respiration rates were measured daily (immediately following shark trials) and subsequently used to correct the slopes observed during Greenland shark trials. Dissolved oxygen concentrations were measured every ten seconds using an HQ40d meter and two LDO101 probes (HACH).

All trials conducted in the Scott Inlet respirometer setup resulted in dissolved oxygen depletions with high R^2^ values (> 0.95). The R^2^ values for the individual studied in the Tremblay Sound respirometer were lower (0.67–0.93), despite depletions being linear (i.e. residuals were evenly scattered around fitted line). This was a result of the large volume of water in the respirometer, the very slow rate of oxygen uptake by the shark, and the level of sensitivity of the dissolved oxygen probe over short sampling intervals. Dissolved oxygen levels decreased at a rate that was too slow to be sensed every ten seconds by our probes, leading to greater spread in the raw data and the lower observed R^2^ values over the short measurement periods when the shark maintained continuous resting or swimming behaviour (roughly 20–60 min). While using a smaller respirometer would have improved the R^2^ value, it would have also impeded the shark’s ability to swim, leading to inflated metabolic rate estimates.

### Calculating oxygen consumption rate

Raw oxygen depletion data was used to estimate mass adjusted metabolic oxygen consumption (MO_2_) according to the following equation:

$$ {\text{MO}}_{{2}} = ({\text{V }} \times \Delta {\text{O}}_{{2}} )/(\Delta {\text{t }} \times {\text{ m}}^{{0.{84}}} ) $$

where (V) is the volume of water in the respirometer (total volume – estimated volume of the shark^52^), (m) is the body mass of the shark adjusted using the interspecies allometric scaling exponent derived here (0.84), and (△O_2_) is the change in oxygen concentration over time (△t)^53^. Shark mass was either measured directly for smaller sharks (n = 2 individuals) or estimated using published Fork length (FL)-Body mass relationship for larger individuals (n = 2; m = 1.109 × 10 − 6 × FL3.41990^54^). The slope of each oxygen depletion trial was adjusted using the slope of a blank trial of equal duration (i.e. slope_[with shark present]_ − slope_[with shark absent]_). In doing so, we accounted for any background respiration occurring in the unfiltered seawater used in the respirometers.

### Interspecies comparison of rRMR in sharks and relative to global teleosts

We conducted a literature search and compiled mean SMR and rRMR estimates for all shark species previously studied via respirometry, excluding data for endothermic species (SI, Table S1). Due to the logistic challenges of measuring metabolic rate in large-bodied sharks, most of these experiments were conducted on juveniles. We estimated the relative contributions of log_10_-mass and temperature on the log_10_-metabolic rate (whole-animal estimates) of sharks using multiple regression analysis. To avoid statistical imbalances arising from some species being overrepresented in the data (i.e. multiple studies on one species and/or multiple estimates derived at different temperatures or masses), data were weighted by species (weight = 1/number of points for a given species). Whereas previous meta-analyses on teleost fish have dealt with this issue by selecting a single representative study for each species in the regression^7,34^, we opted to use weighted points to avoid having to omit studies from the already limited number published for sharks (further details available in SI appendix). The model output provided coefficients “a” and “b” describing the contribution of log_10_-mass and temperature to log_10_ whole-animal SMR/rRMR such that:

$$ {\text{Log}}_{{{1}0}} {\text{SMR}} = {\text{b}}\left( {{\text{Log}}_{{{1}0}} {\text{Mass}}} \right) + {\text{a}}\left( {{\text{Temperature}}} \right) $$

where “b” represents the interspecies allometric scaling exponent for sharks (i.e. SMR ∝ Mass^b^) and where “a” can be used to derive an overall Q_10_ value by calculating metabolic rates (R_1_ and R_2_) at both temperature extremes in our data set (T_1_ and T_2_) using the equation above and holding mass constant, then subsequently plugging these values into the Q_10_ equation below:

$${Q}_{10}= {\left(\frac{{R}_{2}}{{R}_{1}}\right)}^{10/\left({T}_{2}-{T}_{1}\right)}$$

For visualization purposes, we plotted the effect of temperature and mass on metabolic rate separately (Fig. 1). We also extracted high and low allometric scaling exponents from published meta-analyses on teleost fish, as well as intraspecific Q_10_ values for sharks, to use as reference points when assessing our interspecific values.

### Ethical statement

All data collection for the present study adhered to federal and local regulations. Support letters for the project were provided by the local communities of Mittimatalik (Pond Inlet) and Kanngiqtugaapik (Clyde River). All field protocols for Scott Inlet and Tremblay Sound were approved by the University of Windsor’s Animal Care Committee (Animal Utilization Project Proposals #18-01 and #17-12). Fishing licenses were obtained through the Department of Fisheries and Oceans Canada.

## Supplementary information

Supplementary Information.
