# Supplementary material for: A first look at the metabolic rate of Greenland sharks (Somniosus microcephalus) in the Canadian Arctic
Source: Sci Rep. 2020 Nov 9;10:19297. doi: 10.1038/s41598-020-76371-0 (PMC7653932; doi:10.1038/s41598-020-76371-0)
Supplement: Supplementary file 1 — Supplementary Information. [file 41598_2020_76371_MOESM1_ESM.docx]

**SUPPLEMENTARY INFORMATION**

**A First look at the metabolic rate of Greenland sharks (Somniosus microcephalus) in the Canadian Arctic**

Eric Ste-Marie^1*^, Yuuki Y. Watanabe^2^, Jayson M. Semmens^3^, Marianne Marcoux^4^ and Nigel E. Hussey^1^

(1) Department of Integrative Biology, University of Windsor, Windsor, Ontario, N9B 3P4, Canada
(2) National Institute of Polar Research, Tachikawa, Tokyo, 190-8518, Japan
(3) Fisheries and Aquaculture Centre, Institute for Marine and Antarctic Studies, University of Tasmania, Taroona, TAS 7053, Australia

(4) Freshwater Institute Science Laboratory (FWI), Central and Arctic Region, Fisheries and Oceans Canada, Winnipeg, Manitoba, R3T 2N6, Canada

* Corresponding author: Eric Ste-Marie.

**Email:**  [stemari@uwindsor.ca](mailto:stemari@uwindsor.ca)

https://orcid.org/0000-0002-1173-4726

***Fishing & Data collection***

In Tremblay Sound in 2018-2019, Greenland sharks were captured using longlines composed of 6-10 hooks spaced ten metres apart baited with char, seal or narwhal donated by Inuit from subsistence fishing/hunting. Lines were set for 3-8 hours and subsequently pulled to the surface by hand. In Scott Inlet sharks were captured using longlines composed of 50 hooks set for 12 hours baited with squid. Lines were pulled using a motorized winch aboard the MV Kiviuq II. Sharks were not tagged, measured or sampled until after the trials took place to avoid unnecessary stress. Instead, individuals were immediately transferred into the respirometer following capture and allowed to acclimate to their surroundings. Only healthy sharks were used in respirometry trials (i.e. No visible injuries and responsive to a nose touch or tail pinch). In Tremblay Sound (2018), we fished for respirometry sharks nearshore to avoid having to tow animals more than two hundred metres to where our large circular field-respirometer was set up on the beach. Once at the beach, our team used an Extra-Large Shark Carrier (121 Animal Handling Products Ltd, Derbyshire, UK) to rapidly transfer the shark from the fjord to the respirometer (~30 seconds air exposure). A similar protocol was used to release the shark following experimentation. In Scott Inlet (2019), sharks were transferred directly from the longline to our rectangular respirometer aboard the MV Kiviuq II. Following experimentation, sharks were lowered back into the fjord using a makeshift sling.

***Interspecies Metabolic Scaling Model***

We conducted multiple regression analysis using the “lm” (linear model) function in R (version 3.5.2). Since neither mass nor temperature scales linearly with metabolic rate, metabolic rate and mass data were log transformed. Since some species of shark were represented by several data points (i.e. multiple studies per species or multiple estimates at different masses or temperatures), the regression analysis was balanced using the weights argument, with weights for each data point set to the inverse of the total number of data points for that species. Typical weighted regressions also incorporate the variance or standard deviation associated with data points in the model. These values, however, were not available for all species/experiments due to a lack of standardized reporting practices in respirometry studies on sharks. The variance or standard deviation for each study was therefore not included in the regression analysis. Furthermore, most studies only provided a mean mass-specific SMR estimate across individuals of varying mass, without providing the mean mass of individuals used in their study (SI Table 1). In such cases, we assumed that the mean mass of individuals was the midpoint between the max and min masses used in the study (a range of masses was always provided when the mean was not). Additionally, three studies provided mass-adjusted values instead of mass-specific estimates. In such cases, we converted the estimate into the mass-specific equivalent for an individual of average mass. Whole animal metabolic rates were then estimated by multiplying mass-specific metabolic rate by the average mass of an individual used in the study. This undoubtedly increased the error surrounding our model; however, most studies covered a relatively narrow range of masses (~2 kg range on average) which likely limited its overall influence on the output of our analysis covering a range of approximately 126 kg.

**SI Table 1:** Literature derived mass-specific SMR (or rRMR) estimates for sharks.

| **Ref** | **Species** | **Temp (C)** | **Mass avg. (kg)** | **Mass Range (kg)** | **N** | **MO_2_**  **(mgO_2_kg^-1^h^-1^)** | **SD** | **SE** | **Method^b^** |
| --- | --- | --- | --- | --- | --- | --- | --- | --- | --- |
| [1] | Carcharhinus acronotus | 28 | 1 | 0.45-3.51 | 10 | 239.8 |  |  | extrapolated |
| [2] | Carcharhinus leucas | 19.7 | 5 | 3-7 | 9 | 101.4^c^ |  |  | extrapolated |
| [2] | Carcharhinus leucas | 32.5 | 5 | 3-7 | 9 | 304.1^c^ |  |  | extrapolated |
| [3] | Carcharhinus limbatus | 29.4 | 1.25^a^ | 1.03-1.47 | 7 | 246 |  |  | extrapolated |
| [4] | Carcharhinus melanopterus | 29.66 | 1.08 |  | 8 | 100.92 | 11.3 |  | real |
| [5] | Chiloscyllium plagiosum | 24 | 0.6 |  | 7 | 227.8^c^ |  | 14.8 | real |
| [6] | Ginglymostoma cirratum | 23 | 2.65^a^ | 1.3-4 | 5 | 106 |  |  | real |
| [7] | Ginglymostoma cirratum | 22.5 | 8.37 | 5-12.4 | 5 | 26.7^c^ | 8 |  | real |
| [3] | Ginglymostoma cirratum | 23.9 | 8.31^a^ | 5.5-11.12 | 8 | 34.8 | 6 |  | real |
| [3] | Ginglymostoma cirratum | 29.3 | 10.1^a^ | 7.8-12.4 | 8 | 62.9 | 8 |  | real |
| [7] | Ginglymostoma cirratum | 29 | 8.99 | 5.6-12.4 | 6 | 44.1^c^ | 17 |  | real |
| [8] | Heterodontus francisci | 14 | 0.7225 | 0.44-0.94 | 4 | 30.6 |  | 3.4 | real |
| [8] | Heterodontus francisci | 16 | 0.61 | 0.41-0.94 | 10 | 33.9 |  | 2.3 | real |
| [8] | Heterodontus francisci | 20 | 0.59 | 0.41-0.94 | 9 | 44.9 |  | 2.4 | real |
| [8] | Heterodontus francisci | 22 | 0.7 | 0.44-0.94 | 3 | 57.9 |  | 2.7 | real |
| [9] | Heterodontus portusjacksoni | 17 | 1.9 | 1-4.3 | 5 | 116.6^c^ |  | 20.4 | real |
| [9] | Mustelus antarcticus | 17 | 4.35 | 0.9-10.6 | 13 | 103.74^c^ |  | 61.9 | real |
| [4] | Negaprion acutidens | 29.29 | 1.55 |  | 3 | 139.95 | 12.07 |  | real |
| [3] | Negaprion brevirostris | 20.6 | 2.77^a^ | 2.07-3.46 | 20 | 64.1 | 16 |  | real |
| [10] | Negaprion brevirostris | 22 | 1.05^a^ | 0.8-1.3 | 13 | 125 |  | 5.68 | real |
| [11] | Negaprion brevirostris | 25 | 1.39 | 1.11-1.61 | 7 | 152.6 |  | 7.3 | extrapolated |
| [3] | Negaprion brevirostris | 29.5 | 2.35^a^ | 1.74-2.95 | 16 | 168.5 | 23 |  | real |
| [12] | Scyliorhinus canicula | 15 | 0.466^a^ | 0.0029-0.929 | 33 | 42.67 |  |  | real |
| [13] | Scyliorhinus stellaris | 25 | 2.5^a^ |  | 12 | 92 |  |  | real |
| (Present) | Somniosus microcephalus | 4.86 | 40.77 |  | 1 | 12.3 |  |  | real |
| (Present) | Somniosus microcephalus | 5.08 | 33.4 |  | 1 | 9.83 |  |  | real |
| (Present) | Somniosus microcephalus | 3.8 | 126 |  | 1 | 10.63 |  |  | real |
| [14] | Sphyrna lewini | 26 | 0.69 | 0.506-0.927 | 17 | 189 | 15 |  | extrapolated |
| [15] | Sphyrna tiburo | 25 | 1.1^a^ | 0.8-1.4 | 12 | 156 |  |  | extrapolated |
| [16] | Sphyrna tiburo | 28 | 1 |  | 8 | 173.4 |  | 11.3 | paralyzed |
| [17] | Squalus acanthias | 10 | 2.06 | 1.87-2.4 | 18 | 32.4 |  | 2.6 | real |
| [18] | Squalus suckleyi | 6.5 | 2.05^a^ | 1.6-2.5 | 9 | 30.95 | 8.37 |  | real |
| [18] | Squalus suckleyi | 10 | 3.25^a^ | 2.16-4.3 | 9 | 25.62 | 3.3 |  | real |
| [19] | Triakis semifasciata | 16 | 4^a^ | 2.2-5.8 | 5 | 105.3 |  | 35.6 | extrapolated |

^a^ Only a mass range was provided by the source, so this value represents the midpoint between the maximum and minimum mass instead of a true average.

^b^ Method refers to whether the SMR value quoted in the original paper was a direct product of respirometry on a shark that was volitionally at rest (i.e. real), or if it was derived by extrapolating estimates measured while the shark was active to a swim speed of zero (i.e. extrapolated). One study conducted respirometry on chemically immobilized sharks (i.e. paralyzed)

^c^ MO_2_ estimate was presented in original paper as mass-adjusted value. The value presented here was converted into a mass-specific value using the same allometric scaling exponent used by the paper.

**SI Table 2:** Literature derived Q_10_ values for sharks. If multiple values were provided in the original study, only those calculated over an ecologically relevant temperature range are presented here.

| **Ref** | **Shark** | **Q_10_** | **Temp range (°C)** |
| --- | --- | --- | --- |
| [2] | Carcharhinus leucas | 1.88 | 19.7-32.5 |
| [3] | Carcharhinus limbatus | 2.67 | 21.6-29.4 |
| [20] | Carcharhinus plumbeus | 2.5 | 24-28 |
| [21] | Chiloscyllium plagiosum | 2.7 | 15-30 |
| [7] | Ginglymostoma cirratum | 2.42 | 23-30 |
| [3] | Ginglymostoma cirratum | 2.99 | 23.9-29.3 |
| [8] | Heterodontus francisci | 2.01 | 14-22 |
| [3] | Negaprion brevirostrus | 2.96 | 20.6-29.5 |
| [22] | Scyliorhinus canicula | 2.1 | 7-17 |
| [14] | Sphyrna lewini | 1.34 | 21-29 |
| [23] | Sphyrna tiburo | 2.34 | 20-30 |
| [24] | Squalus acanthias | 2.59 | 7.5-12 |
| [25] | Triakis semifasciata | 2.51 | 12-24 |

**SI Table 3:** Individual trial estimates (mass-adjusted) for the Greenland sharks studied via respirometry. rRMR estimates are presented in white and aRMR estimates are in blue. Red estimates are for trials where the shark exhibited sporadic movement throughout the trial making it impossible to estimate true rRMR or aRMR. Durations for each measurement interval are provided in minutes (min).

|  |  | **Trial Estimates (mgO_2_kg^-1^h^-0.84^)** | | | | | | | | | | | | | | | | | | | | | | | | | | |  |
| --- | --- | --- | --- | --- | --- | --- | --- | --- | --- | --- | --- | --- | --- | --- | --- | --- | --- | --- | --- | --- | --- | --- | --- | --- | --- | --- | --- | --- | --- |
| **Shark ID** | **Respirometer** | **1** | | |  | | **2** | | | |  | | **3** | | | |  | | **4** | | | |  | | **5** | | | |  |
|  |  | **MO_2_** | **min** | **R^2^** | |  | | **MO_2_** | **min** | **R^2^** | |  | | **MO_2_** | **min** | **R^2^** | |  | | **MO_2_** | **min** | **R^2^** | |  | | **MO_2_** | **min** | **R^2^** | |
| 1 | Circular | 26.01 | 30 | 0.790 | |  | | 30.96 | 20 | 0.757 | |  | | 16.26 | 40 | 0.673 | |  | | 25.89 | 60 | 0.935 | |  | | 24.10 | 33 | 0.760 | |
| 2 | Rectangular | 24.97 | 29 | 0.994 | |  | | 19.22 | 30 | 0.955 | |  | | 33.27 | 60 | 0.997 | |  | | 31.29 | 60 | 0.999 | |  | | 22.67 | 15 | 0.975 | |
| 3 | Rectangular | 38.32 | 60 | 0.994 | |  | | 42.66 | 60 | 0.999 | |  | | 40.38 | 60 | 0.999 | |  | |  |  |  | |  | |  |  |  | |
| 4 | Rectangular | 16.00 | 60 | 0.996 | |  | | 17.25 | 60 | 0.998 | |  | | 18.12 | 60 | 0.998 | |  | | 17.57 | 60 | 0.999 | |  | |  |  |  | |

**SI REFERENCES**

1. Carlson, J. K., Palmer, C. L. & Parsons, G. R. Oxygen consumption rate and swimming efficiency of the blacknose shark, Carcharhinus acronotus. *Copeia* 34–39 (1999).

2. Lear, K. O. *et al.* Divergent field metabolic rates highlight the challenges of increasing temperatures and energy limitation in aquatic ectotherms. *Oecologia* (2020).

3. Lear, K. O. *et al.* Correlations of metabolic rate and body acceleration in three species of coastal sharks under contrasting temperature regimes. *J. Exp. Biol.* **220**, 397–407 (2017).

4. Bouyoucos, I. A., Weideli, O. C., Planes, S., Simpfendorfer, C. A. & Rummer, J. L. Dead tired: evaluating the physiological status and survival of neonatal reef sharks under stress. *Conserv. Physiol.* **6**, (2018).

5. Di Santo, V. & Bennett, W. A. Effect of rapid temperature change on resting routine metabolic rates of two benthic elasmobranchs. *Fish Physiol. Biochem.* **37**, 929–934 (2011).

6. Fournier, R. W. The metabolic rates of two species of benthic elasmobranchs, nurse sharks & southern stingrays. (1996).

7. Whitney, N. M., Lear, K. O., Gaskins, L. C. & Gleiss, A. C. The effects of temperature and swimming speed on the metabolic rate of the nurse shark (Ginglymostoma cirratum, Bonaterre). *J. Exp. Mar. Bio. Ecol.* **477**, 40–46 (2016).

8. Luongo, S. M. & Lowe, C. G. Seasonally acclimated metabolic Q10of the California horn shark, Heterodontus francisci. *J. Exp. Mar. Bio. Ecol.* **503**, 129–135 (2018).

9. Molina, J. M., Finotto, L., Walker, T. I. & Reina, R. D. The effect of gillnet capture on the metabolic rate of two shark species with contrasting lifestyles. *J. Exp. Mar. Bio. Ecol.* **526**, 151354 (2020).

10. Bushnell, P. G., Lutz, P. L. & Gruber, S. H. The metabolic rate of an active, tropical elasmobranch, the lemon shark (Negaprion brevirostris). *Exp. Biol* **48**, (1989).

11. Scharold, J. & Gruber, S. H. Telemetered heart rate as a measure of metabolic rate in the lemon shark, Negaprion brevirostris. *Copeia* 942–953 (1991).

12. Sims, D. W. The effect of body size on the standard metabolic rate of the lesser spotted dogfish. *J. Fish Biol.* **48**, 542–544 (1996).

13. Piiper, J., Meyer, M., Worth, H. & Willmer, H. Respiration and circulation during swimming activity in the dogfish Scyliorhinus stellaris. *Respir. Physiol.* **30**, 221–239 (1977).

14. Lowe, C. Metabolic rates of juvenile scalloped hammerhead sharks (Sphyrna lewini). *Mar. Biol.* **139**, 447–453 (2001).

15. Carlson, J. K. The physiological ecology of the bonnethead shark, Sphyrna tiburo, blacknose shark, Carcharhinus acronotus, and Florida smoothhound shark, Mustelus norrisi: Effects of dissolved oxygen and temperature. (University of Mississippi, 2000).

16. Carlson, J. K. & Parsons, G. R. Respiratory and hematological responses of the bonnethead shark, Sphyrna tiburo, to acute changes in dissolved oxygen. *J. Exp. Mar. Bio. Ecol.* **294**, 15–26 (2003).

17. Brett, J. R. & Blackburn, J. M. Metabolic Rate and Energy Expenditure of the Spiny Dogfish, Squalus acanthias. *J. Fish. Res. Board Canada* **35**, 816–821 (1978).

18. Hanson, D. & Johansen, K. Relationship of gill ventilation and perfusion in Pacific dogfish, Squalus suckleyi. *J. Fish. Board Canada* **27**, 551–564 (1970).

19. Scharold, J., Lai, N. C., Lowell, W. R. & Graham, J. B. Metabolic rate, heart rate, and tailbeat frequency during sustained swimming in the leopard shark Triakis semifasciata. *Exp. Biol.* **48**, 223–230 (1989).

20. Dowd, W., Brill, R. W., Bushnell, P. G. & Musick, J. A. Standard and routine metabolic rates of juvenile sandbar sharks (Carcharhinus plumbeus), including the effects of body mass and acute temperature change. *Fish. Bull.* (2006).

21. Tullis, A. & Baillie, M. The metabolic and biochemical responses of tropical whitespotted bamboo shark Chiloscyllium plagiosum to alterations in environmental temperature. *J. Fish Biol.* **67**, 950–968 (2005).

22. Butler, P. J. & Taylor, E. W. The effect of progressive hypoxia on respiration in the dogfish (Scyliorhinus canicula) at different seasonal temperatures. *J. Exp. Biol.* **63**, 117–130 (1975).

23. Carlson, J. K. & Parsons, G. R. Seasonal differences in routine oxygen consumption rates of the bonnethead shark. *J. Fish Biol.* **55**, 876–879 (1999).

24. Giacomin, M., Schulte, P. M. & Wood, C. M. Differential Effects of Temperature on Oxygen Consumption and Branchial Fluxes of Urea, Ammonia, and Water in the Dogfish Shark (Squalus acanthias suckleyi). *Physiol. Biochem. Zool.* **90**, 627–637 (2017).

25. Miklos, P., Katzman, S. M. & Cech, J. J. Effect of temperature on oxygen consumption of the leopard shark, Triakis semifasciata. *Environ. Biol. Fishes* **66**, 15–18 (2003).
